# Exercise Overrides Blunted Hypoxic Ventilatory Response in Prematurely Born Men

**DOI:** 10.3389/fphys.2019.00437

**Published:** 2019-04-16

**Authors:** Tadej Debevec, Vincent Pialoux, Grégoire P. Millet, Agnès Martin, Minca Mramor, Damjan Osredkar

**Affiliations:** ^1^Faculty of Sport, University of Ljubljana, Ljubljana, Slovenia; ^2^Department of Automation, Biocybernetics and Robotics, Jožef Stefan Institute, Ljubljana, Slovenia; ^3^Laboratoire Interuniversitaire de Biologie de la Motricité, Claude Bernard University Lyon 1, Villeurbanne, France; ^4^Institut Universitaire de France, Paris, France; ^5^Faculty of Biology and Medicine, Institute of Sport Sciences of the University of Lausanne, University of Lausanne, Lausanne, Switzerland; ^6^Master BioSciences, Ecole Normale Supérieure de Lyon, Université Claude-Bernard Lyon 1, Lyon, France; ^7^Department of Pediatric Emergency, University Children’s Hospital Ljubljana, Ljubljana, Slovenia; ^8^Department of Pediatric Neurology, University Children’s Hospital Ljubljana, Ljubljana, Slovenia

**Keywords:** pre-term birth, hypoxia, ventilatory response, oxidative stress, exercise capacity

## Abstract

**Purpose:**

Pre-term birth provokes life-long anatomical and functional respiratory system sequelae. Although blunted hypoxic ventilatory response (HVR) is consistently observed in pre-term infants, it remains unclear if it persists with aging and, moreover, if it influences hypoxic exercise capacity. In addition, it remains unresolved whether the previously observed prematurity-related alterations in redox balance could contribute to HVR modulation.

**Methods:**

Twenty-one prematurely born adult males (gestational age = 29 **±** 4 weeks], and 14 age matched controls born at full term (gestational age = 39 **±** 2 weeks) underwent three tests in a randomized manner: (1) hypoxia chemo-sensitivity test to determine the resting and exercise poikilocapnic HVR and a graded exercise test to volitional exhaustion in (2) normoxia (F_i_O_2_ = 0.21), and (3) normobaric hypoxia (F_i_O_2_ = 0.13) to compare the hypoxia-related effects on maximal aerobic power (MAP). Selected prooxidant and antioxidant markers were analyzed from venous samples obtained before and after the HVR tests.

**Results:**

Resting HVR was lower in the pre-term (0.21 **±** 0.21 L ⋅ min^-1^ ⋅ kg^-1^) compared to full-term born individuals (0.47 **±** 0.23 L ⋅ min^-1^ ⋅ kg^-1^; *p* < 0.05). No differences were noted in the exercise HVR or in any of the measured oxidative stress markers before or after the HVR test. Hypoxia-related reduction of MAP was comparable between the groups.

**Conclusion:**

These findings indicate that blunted resting HVR in prematurely born men persists into adulthood. Also, active adults born prematurely seem to tolerate hypoxic exercise well and should, hence, not be discouraged to engage in physical activities in hypoxic environments. Nevertheless, the blunted resting HVR and greater desaturation observed in the pre-term born individuals warrant caution especially during prolonged hypoxic exposures.

## Introduction

An estimated 10% of infants are born prematurely each year (Purisch and Gyamfi-Bannerman, 2017). Pre-term birth and associated medical interventions hinder lung development and can result in life-long anatomical and functional sequelae of the respiratory (and many other) systems (Lovering et al., 2014). Numerous studies demonstrate persistence of various respiratory limitations and symptoms in the pre-term born individuals during the course of maturation (McLeod et al., 1996; Palta et al., 2001; Vrijlandt et al., 2007).

It has previously been shown in both human and rodent studies that perinatal hyperoxia, often associated with treatment of premature newborns, can provoke significant alterations in cardiorespiratory control which can subsequently affect ventilatory responses in normoxic and hypoxic conditions (Bisgard et al., 2003; Bavis, 2005). In addition, hyperoxia-related carotid chemoreceptor dysfunction also seems to result in abnormal ventilatory responses of prematurely born individuals (Bates et al., 2014). While the overall consequences of these cardio-respiratory dysfunctions are not well established, the blunted hypoxic ventilatory response (HVR), consistently reported in pre-term born infants (Katz-Salamon and Lagercrantz, 1994), might have important implications. Indeed, although limited, recent evidence indicates that the reduced HVR can persist with aging (Bates et al., 2014). Recent evidence also suggests that prematurely born adults could exhibit higher oxidative stress levels (Filippone et al., 2012) as compared to their full-term born counterparts. Indeed, as recently reviewed by Martin et al. (2018) preterm-birth related higher oxidative stress levels may persist into adulthood and consequently negatively influence a number of physiological processes. Given that oxidative stress levels have previously been shown to importantly modulate HVR (Pialoux et al., 2009a,b) it seems important to investigate this relationship as it might give us mechanistical insight into the HVR modulation. The above noted respiratory and systemic sequelae may importantly influence exercise capacity as well as the ability of pre-term born individuals to adapt to altitude/hypoxic environment.

Reduced overall exercise capacity of the pre-term cohort is clearly established by numerous observational and interventional trials (Rogers et al., 2005; Vrijlandt et al., 2006; Saigal et al., 2007; Lovering et al., 2013; Svedenkrans et al., 2013). While the consequences of pre-term birth are rather well characterized for normoxic exercise, the potential influence of prematurity on hypoxic exercise tolerance remains largely unclear. Very few studies to-date compared hypoxic exercise capacities between the pre-term and full term born individuals. While Farrell et al. (2015) found significantly lower absolute power outputs in pre-term vs. full-term born individuals with relatively low aerobic capacity during graded exercise in normoxia, surprisingly, no such difference was noted during hypoxic exercise. Also, Duke et al. (2014) did not find any significant differences between the pre-term born individuals with low diffusion capacity and healthy full term born individuals in the relative cardio-respiratory and pulmonary gas exchange responses to graded exercise in neither normoxia nor hypoxia. It is important to note that all of the above studies have scrutinized the effects of hypoxia in cohorts of untrained and inactive pre-term born individuals. Accordingly, there is an obvious lack of studies investigating hypoxic exercise responses in healthy, aerobically fit and active pre-term born individuals which are most likely to engage in high-altitude activities. Therefore, and given an ever-increasing number of individuals who visit high-altitude regions for sports participation or recreation (Richalet et al., 2012), it is crucial to determine, identify, and subsequently reduce the hypoxia-related clinical risks in the active pre-term born individuals.

The purpose of this study was to determine the effects of acute hypoxia on poikilocapnic HVR and exercise performance in healthy, active pre-term born adult males and compare them to their age and aerobic capacity matched counterparts born at full-term. We hypothesized that: (i) pre-term born adults will exhibit lower HVR both, at rest and during moderate exercise and (ii) hypoxia will induce significantly greater reduction in maximal aerobic power (MAP) in the active pre-term vs. full-term born adults. Finally, we also tested the potential differences in systemic oxidative stress between the pre-term vs. full-term born individuals.

## Materials and Methods

### Participants

The recruitment procedure for the pre-term born participants was based on the National pre-term birth register established and maintained at the Clinical Centre in Ljubljana, Slovenia. The following inclusion criteria were used for the pre-term (gestational age ≤32 weeks; gestational body mass ≤1500 g; hyperoxic treatment at birth) and full-term (gestational age ≥38 weeks; gestational body mass ≥2500 g) born individuals. Exclusion criteria included permanent altitude residence (≥1000 m), cardiopulmonary, hematological and/or kidney disorders, regular medication use, smoking and altitude/hypoxia exposure (≥2000 m) within the last moth prior to the study. Upon initial medical record screening and individual telephone interviews, twenty-one prematurely born adult males [gestational age = 29 ± 4 weeks; gestational body mass = 1264 ± 297 g; (mean ± SD)] and fourteen age and aerobic capacity matched controls born at full term (gestational age = 39 ± 2 weeks; gestational body mass = 3671 ± 499 g) were recruited. Baseline characteristics of both groups are detailed in Table 1. The participants of both groups were recreationally active (≥2 aerobic exercise sessions per week) and were provided with extensive written and verbal explanation of the experimental procedures as well as potential risks involved. All participants completed health and activity questionnaires and signed a written informed consent before the study onset.

**Table 1:** Baseline characteristics of the pre-term and full-term born participants.

	Pre-term born	Full-term born
N	21	14
Age (years)	21 ± 1	22 ± 2
Height (cm)	175 ± 8	180 ± 5^∗^
Body mass (kg)	69 ± 8	73 ± 6
Body mass at birth (g)	1264 ± 297	3671 ± 499^∗∗^
Gestational age (Weeks)	29 ± 4	39 ± 2^∗∗^
BMI (kg ⋅ m^2^)	22 ± 3	22 ± 1
% Body fat	19 ± 8	17 ± 5
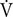 O_2peak_ (mL ⋅ kg^-1^ ⋅ min^-1^)	48.5 ± 6.3	51.7 ± 4.5
FVC (L) [% predicted]	5.1 ± 0.6 [97%]	5.5 ± 0.8 [97%]
FEV_1_ (L) [% predicted]	4.3 ± 0.6 [96%]	4.7 ± 0.8 [98%]
FEV_1_/FVC (%)	0.85 ± 0.06	0.86 ± 0.07
Red blood cells (10^-12^ ⋅ L^-1^)	5.2 ± 0.3	5.0 ± 0.4
Hematocrit (%)	44 ± 4	44 ± 4
Hemoglobin (g ⋅ L^-1^)	155 ± 12	153 ± 10

*Data are presented as mean ± SD. BMI, body mass index; 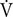
O_*2peak*_, peak oxygen uptake during normoxic exercise; FVC, forced vital capacity; FEV_*1*_, forced expired volume in 1 s; FEV_*1*_/FVC, ratio of forced expired volume and forced vital capacity. ^∗^*p* < 0.05, ^∗∗^*p* < 0.01 denotes significant differences as compared to pre-term born individuals*.

### Study Overview

The study comprised of familiarization visit during which all participants underwent a medical pre-screening, baseline anthropometric measurement and lung function assessment and three experimental visits on separate occasions during which the participants underwent the following tests in a randomized and counterbalanced manner: (1) Hypoxia chemo-sensitivity test to determine the resting and exercise poikilocapnic HVR and a graded exercise test to volitional exhaustion in (2) Normoxia (F_i_O_2_ = 0.21), and (3) Normobaric hypoxia (F_i_O_2_ = 0.13) to compare hypoxia-related effects on MAP. Both exercise tests were performed at the same time of the day. The participants were instructed to consume the same standardized breakfast and to avoid alcohol, caffeine and exercise for at least 24 h prior to each visit. The study protocol was pre-registered at ClinicalTrials.gov (NCT02780908), approved by the National Committee for Medical Ethics at the Ministry of Health of the Republic of Slovenia (0120-101/2016-2) and conducted according to the guidelines of the Declaration of Helsinki.

### Anthropometry and Lung Function Test

Baseline measurements of body mass and body height were performed using a stadiometer-scale (Libela ELSI, Celje, Slovenia). The % body fat was calculated using the Jackson and Pollock (1978) equation from nine skin-fold measurement sites (triceps, subscapular, chest, suprailiac, abdominal, thigh (3 sites) and inguinal). The lung function testing was performed using the pneumotachograph (Cardiovit AT-2plus, Schiller, Baar, Switzerland) in line with the established criteria (Miller et al., 2005). The device was calibrated prior to each test using a 3-L syringe. Each test was performed three consecutive times and the highest of the three values of forced vital capacity (FVC) and forced expiratory volume in 1 s (FEV_1_) were recorded and subsequently used to calculate the FEV_1_/FVC ratio. The percentage predicted FVC and FEV_1_ values were calculated based on the GLI ERS Task Force equations (Quanjer et al., 2012).

### Hypoxia Sensitivity Test

The hypoxia chemo-sensitivity test (Richalet et al., 2012; Bourdillon et al., 2014) was used to assess the poikilocapnic HVR, during both rest and moderate exercise. The protocol was conducted on a cycloergometer (Ergo Bike Premium, Daum electronics, Fürth, Germany) and comprised the following four 4-min steps performed in sequential order: (1) resting normoxia (RN); (2) resting hypoxia (RH); (3) hypoxic exercise (EH); and (4) normoxic exercise (EN). During the test, the participants breathed through an oro-nasal mask (Vmask^TM^, Hans Rudolph, Shawnee, KS, United States) connected to a calibrated metabolic cart and pneumotachograph (Quark CPET; Cosmed, Rome, Italy) to continuously monitor breath-by-breath ventilation levels and gas exchange. Throughout both hypoxic HVR test steps, the participants inspired a humidified hypoxic gas mixture (F_i_O_2_ = 0.11, P_i_O_2_ = 77 mmHg) from a 200-L Douglas bag via a two-way low resistance valve (2700 NRBV; Hans Rudolph Inc., Shawnee, KS, United States). In line with the standard hypoxia chemo-sensitivity test protocol (Richalet et al., 2012; Bourdillon et al., 2014) the external power output on the cycle ergometer was set at an appropriate level to elicit a heart rate (HR) of ∼130 beats ⋅ min^-1^ at the start of the first exercise phase, and maintained constant thereafter. Capillary O_2_ saturation (SpO_2_) and HR were measured using a finger oximetry device (BCI 3301; Nellcor, Boulder, CO) and a portable heart rate monitor (Polar S810i, Kempele, Finland), respectively. The averaged data of the last minute of each testing step (RN, RH, EH, and EN) was employed for subsequent calculation of the resting (HVRr) and exercise (HVRe) poikilocapnic HVRs performed using the following equations (Lhuissier et al., 2012; Bourdillon et al., 2014):

Desaturation at rest: SpO_2_r = SpO_2_rn – SpO_2_rh (%)

Desaturation at exercise: SpO_2_e = SpO_2_en - SpO_2_eh (%)

Ventilation changes at rest: 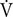
Er = 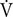
Erh - 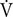
Ern (L ⋅ min^-1^);

Ventilation changes at exercise: 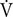
Ee = 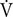
Eeh - 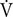
Een (L ⋅ min^-1^);

Hypoxic ventilatory response at rest: HVRr = 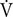
Er ⋅ SpOr^-1^ ⋅ BM^-1^ (L ⋅ min^-1^ ⋅ kg^-1^);

Hypoxic Ventilatory response at exercise: HVRe = 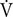
Ee ⋅ SpOe^-1^ ⋅ BM^-1^ (L ⋅ min^-1^ ⋅ kg^-1^);


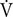
E, minute ventilation; BM, body mass; r, rest; e, exercise; n, normoxia; and h, hypoxia.

### Exercise Tests

On two separate occasions, the participants performed two graded exercise tests in a randomized and single-blinded manner on an electromagnetically braked cycloergometer (Ergo Bike Premium, Daum electronics, Fürth, Germany). The protocol commenced with a rest period (5-min in normoxic condition during all normoxic test and 5-min normoxic and 5-min hypoxic during all hypoxic exercise tests), followed by a 5-min warm up at 60 W. Thereafter the workload was increased for 40 W ⋅ 2 min^-1^ until volitional exhaustion. The participants were required to maintain a cadence ≥60 revolutions ⋅ min^-1^ throughout the whole test. The test was terminated when the participants were unable to maintain the assigned cadence. Strong verbal encouragement from the experimental personnel was applied during the later stages of each test. The exercise tests were always performed at the same time of the day under standardized environmental conditions with participants seated through the whole test duration. During both tests, the participants breathed through an oro-nasal mask (Vmask^TM^, Hans Rudolph, Shawnee, KS, United States), connected to a two-way low resistance valve (2700 NRBV; Hans Rudolph Inc., Shawnee, KS, United States). On one occasion the valve was connected to a 200-L Douglas bag containing ambient air (normoxia (placebo); F_i_O_2_ = 0.21; P_i_O_2_ = 147 mmHg). On another occasion, the Douglas bag contained normobaric hypoxic gas mixture (hypoxia; F_i_O_2_ = 0.13; P_i_O_2_ = 91 mmHg). Breath-by-breath gas exchange and ventilation responses during resting and exercise phases were measured using a calibrated metabolic cart (Quark CPET, Cosmed, Rome, Italy). Peak O_2_ uptake (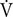
O_2peak_), was defined as the highest 60-s average of O_2_ uptake during the test. The same time average was used to determine the cardiorespiratory values and ratings of perceived exertion at volitional exhaustion. The resting values were derived from the 60-s averages of the last minute of the rest period during the normoxic exercise tests and the last minute of the resting period in hypoxic condition during the hypoxic exercise tests. MAP was calculated using the following equation as detailed previously (Debevec et al., 2010): MAP (W) = W_compl_ + [(*t* ⋅ 120^-1^) ⋅ 40].

W_compl_ corresponds to the last completed workload and *t* corresponds to the number of seconds during final uncompleted workload.

### Blood Sampling and Biochemical Analyses

Venous blood samples were obtained before and immediately after the hypoxia chemo-sensitivity test to determine the plasma markers of oxidative stress [advanced oxidation protein products (AOPP), malondialdehyde (MDA) and nitrotyrosine] and antioxidant status [superoxide dismutase (SOD), ferric-reducing antioxidant power (FRAP), glutathione peroxidase (GPX) and catalase]. Five milliliters samples were, on each occasion, obtained from the antecubital vein and collected in the EDTA tubes (Vacutainer K2E, Becton Dickinson, Plymouth, United Kingdom). The venous blood was immediately centrifuged (10-min at 3500 rpm; 4C) and the obtained plasma frozen to -80°C in small (400-μL) aliquots for subsequent blinded analysis performed within 6 months. An additional 3 mL of whole blood was obtained before the test and analyzed immediately using the cytochemical impedance method (Pentra120; Horiba ABX Diagnostics, Montpellier, France; coefficient of variation (CV) <2%) to determine the baseline hemoglobin levels.

The prooxidant/antioxidant biochemical analysis was performed using TECAN Infinite 2000 plate reader (Männedorf, Switzerland). Briefly, AOPP plasma concentration was read at 340 nm and is expressed as μmol ⋅ L^-1^ of chloramine-T equivalent as previously described (Witko-Sarsat et al., 1996). The intra-assay analysis CV was 5.4%.

Malondialdehyde concentrations were determined via extracting the pink chromogen formed from the reaction between MDA and 2-thiobarbituric acid at 100°C and measuring its absorbance at 532 nm by spectrophotometry using 1,1,3,3-tetraethoxypropan as standard (Ohkawa et al., 1979). The intra-assay analysis CV was 2.2%.

Concentrations of plasma nitrotyrosine, as peroxynitrite (ONOO^-^) protein nitration end product, were measured as previously described (Galinanes and Matata, 2002) via a competitive ELISA assay using an anti-nitrotyrosine primary antibody produced in rabbit (Sigma-Aldrich, Saint-Louis, MI, United States; 1:10,000, 1.5 h at room temperature). The fixation of the anti-rabbit IgG-HRP-conjugate secondary antibody (Invitrogen, Carlsbad, CA, United States; 1:100, 1 h at room temperature) was then read by spectrophotometry at 450 nm. A bovine serum albumin solution at different concentrations was used as standard. The intra-assay analysis CV was 6.8%.

The plasma SOD activity was measured using the method Beauchamp and Fridovich (1971), slightly modified by Oberley and Spitz (1984) by spectrophotometry using the degree of inhibition of the reaction between superoxide radicals, produced by a hypoxanthine – xanthine oxidase system, and nitroblue tetrazolium. The intra-assay analysis CV was 5.6%.

FRAP is determined using the method of Benzie and Strain (1996) by measuring the ability of the plasma to reduce ferric into ferrous iron. FRAP of plasma was calculated using an aqueous solution of known Fe^2+^ concentration (FeSO_4_, 7H_2_O_2_) as standard at a wavelength of 593 nm via spectrophotometry at a controlled temperature (37°C). The intra-assay analysis CV was 2.9%.

Glutathione peroxidase activity in the plasma was assessed by the modified method of Paglia and Valentine (1967) by spectrophotometry at a wavelength of 340 nm the rate of oxidation of NADPH into NADP^+^ after addition of glutathione reductase (GR), reduced glutathione (GSH) and NADPH, using H_2_O_2_ as a substrate. The intra-assay analysis CV was 4.6%.

Catalase activity was determined by the method of Johansson and Borg (1988) using formaldehyde as a standard and hydrogen peroxide (H_2_O_2_) as a substrate. The catalase activity was subsequently determined by the formation rate of formaldehyde read by spectrophotometry induced by the reaction of methanol and H_2_O_2_ using catalase as enzyme. The intra-assay analysis CV was 3.1%.

### Statistical Analysis

Data are presented as mean **±** SD unless otherwise indicated. Dependent-samples Student’s *t*-test was used to compare participants’ baseline characteristics. Two-way unbalanced ANOVA (pre-term vs. full-term group × rest vs. exercise) was used to compare the resting and exercising HVR of the pre-term and full-term born participants. For the graded exercise test variables analysis, a two-way unbalanced ANOVA with repeated measures (Hypoxic vs. Normoxic and/or Pre vs. Post) was employed to test for interactions and main effects for all exercise responses and prooxidant/antioxidant markers. If a significant F-ratio for the main effect or an interaction was observed a Tukey’s HSD *post hoc* test was used to elucidate specific differences. Pearson’s correlation coefficient was used to explore the bivariate correlations between the changes in MAP and HVR and the measured oxidative stress and antioxidant markers. Normality of distribution was confirmed using the Kolmogorov-Smirnov test. *A priori* power analysis using G^∗^Power software (version 3.1.9.3) was conducted to determine the appropriate sample size. Based on the data from previous reports (Bates et al., 2014; Duke et al., 2014; Farrell et al., 2015) 13 participants per group were required to yield the targeted analysis power of ≥0.8 at α = 0.05 for the main outcomes (HVR and MAP). The *Post hoc* analysis indicated the statistical power of 0.91 and 0.93 for the HVR and MAP, respectively with the employed sample size (Pre-term *N* = 21; Full-term *N* = 14). The level of significance was defined *a priori* at *P* < 0.05 and the analyses performed using Statistica 12.0 (StatSoft, Tulsa, United States).

## Results

### Group Characteristics

As noted in Table 1, the groups were matched for all of the baseline anthropometric characteristics except for the body height, with the pre-term born participants being significantly shorter that the full-term ones (*p* = 0.04). Importantly the groups had comparable indexes of pulmonary capacity (FEV_1_ and FEV_1_/FVC ratio), hematological parameters and peak aerobic capacity.

### Hypoxic Ventilatory Response

**Figure 1:**
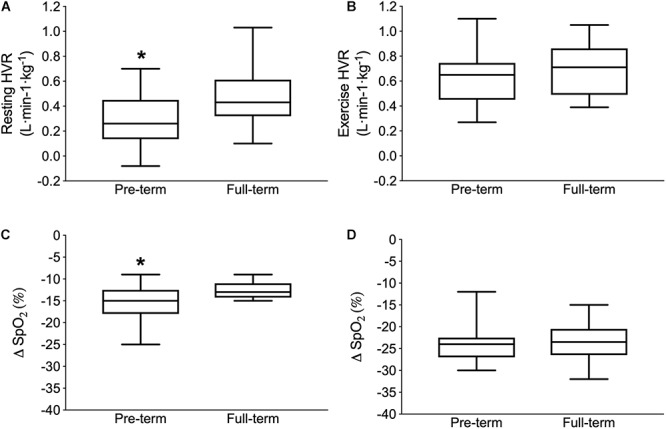
Poikilocapnic hypoxic ventilatory response (HVR) at rest (Upper left panel **A**) and during exercise (Upper right panel **B**) as well as the capillary oxygen desaturation (Δ SpO_2_ from normoxia to hypoxia) during the resting (Lower left panel **C**) and exercise (Lower right panel **D**) parts of the hypoxia chemo-sensitivity test. Boxes depict the median and first and third interquartile ranges, with whiskers indicating maximum and minimum responses. ^∗^Indicates significantly different values compared to full-term born individuals (*p* < 0.05).

The resting HVR was significantly lower in the pre-term than in full-term born individuals (0.21 ± 0.21 vs. 0.47 ± 0.23 L ⋅ min^-1^ ⋅ kg^-1^; *p* = 0.03; Figure 1A). In contrast, no differences were noted in the exercise HVR (pre-term: 0.62 ± 0.23 vs. full-term: 0.70 ± 0.20 L ⋅ min^-1^ ⋅ kg^-1^; *p* = 0.50; Figure 1B). In comparison to the full-term, the pre-term born participants exhibited a significantly greater hypoxia-related desaturation during rest [(ΔSpO_2_) pre-term: -15% ± 4% vs. full-term: -12% ± 2%; *p* = 0.028; Figure 1C], but not during the exercising part of the hypoxic chemo-sensitivity test (pre-term: -24% ± 5% vs. full-term: -24% ± 5%; *p* = 0.79; Figure 1D). No differences between the groups were noted in the cardiac outcomes derived from the hypoxia sensitivity test (*p* = 0.18).

### Exercise Responses

**Figure 2:**
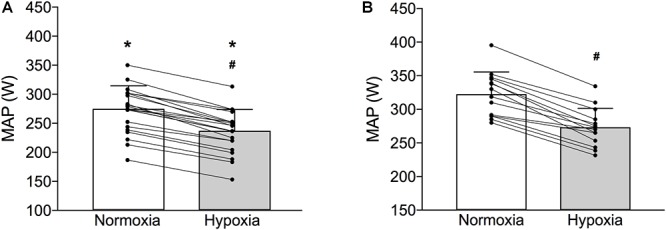
Maximal aerobic power (MAP) of the pre-term (Panel **A**) and full-term (Panel **B**) born individuals obtained during the graded exercise test in normoxia and normobaric hypoxia (bars depict means ± SD whereas lines depict individual changes). ^∗^Indicates significantly different MAP values compared to full-term born individuals in both normoxia and hypoxia (*p* < 0.01); #Indicates significant within-group differences normoxic and hypoxic MAP values (*p* < 0.01).

A significantly lower absolute MAP was observed in the pre-term than in the full-term individuals in both normoxic (pre-term: 272 ± 39 W; full-term:322 ± 33 W *p* < 0.001) and hypoxic (pre-term: 235 ± 36 W; full-term:273 ± 28 W *p* < 0.001) conditions (Figure 2). However, the hypoxia-induced reduction of MAP was comparable between the two groups (pre-term: -8.6 ± 0.4 vs. full-term: -8.5 ± 0.5%; *p* = 0.35; Figure 2). It is also of note that the measured cardio-respiratory parameters at rest and at volitional exhaustion, detailed in Table 2, during both, normoxic and hypoxic graded tests were similar between the two groups. The only exception being a significantly higher V_T_ observed at exhaustion in the full-term as compared to the pre-term born individuals during the normoxic exercise (*p* = 0.046).

**Table 2:** Cardiorespiratory values and ratings of perceived exertion at rest and at volitional exhaustion during the graded exercise tests performed in normoxic (F_i_O_2_ = 0.21) and normobaric hypoxic (F_i_O_2_ = 0.13) condition.

	Normoxia				Hypoxia			
	Pre-term		Full-term		Pre-term		Full-term	
	Rest	Exhaustion	Rest	Exhaustion	Rest	Exhaustion	Rest	Exhaustion
SpO_2_ (%)	97.1 ± 0.9	94.6 ± 0.5	97.4 ± 0.9	93.8 ± 0.6	86.7 ± 2.8	75.7 ± 1.2	87.8 ± 2.0	75.2 ± 1.4
HR (beats ⋅ min^-1^)	83 ± 14	183 ± 3	83 ± 16	183 ± 3	95 ± 12	182 ± 3	95 ± 11	184 ± 3
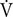 O_2peak_ (mL ⋅ kg^-1^ ⋅ min^-1^)	/	48.5 ± 6.3	/	51.7 ± 4.5	/	42.1 ± 7.1	/	45.0 ± 4.1
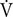 _E_ (L ⋅ min^-1^)	13 ± 2	123 ± 6	12 ± 3	126 ± 5	17 ± 4	119 ± 6	16 ± 3	129 ± 7
V_T_ (L)	0.9 ± 0.3	2.70 ± 0.08	0.9 ± 0.3	2.98 ± 0.09^∗^	1.10 ± 0.24	2.62 ± 0.07	1.22 ± 0.31	2.75 ± 0.11
f_R_ (breaths ⋅ min^-1^)	16 ± 3	46 ± 2	15 ± 4	43 ± 2	16 ± 3	46 ± 2	15 ± 4	48 ± 2
RPE_leg_ (a.u.)	6.1 ± 0.2	19.0 ± 0.3	6 ± 0	18.6 ± 0.4	6.3 ± 0.8	18.5 ± 0.3	6.2 ± 0.8	18.1 ± 0.5
RPE_dys_ (a.u.)	6.1 ± 0.2	17.3 ± 0.5	6.1 ± 0.2	17.9 ± 0.5	6.9 ± 1.5	17.6 ± 0.4	7.4 ± 2.0	18.3 ± 0.3

*Data are presented as mean ± SD. SpO_*2*_, capillary O_*2*_ saturation; HR, heart rate; 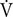
O_*2peak*_, peak oxygen uptake; 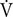
_*E*_, minute ventilation; V_*T*_, tidal volume; f_*R*_, respiratory frequency; RPE_*leg*_, reported ratings of perceived exertion for the legs; RPE_*dys*_, reported ratings of perceived exertion for dyspnea. ^∗^*p* < 0.05 denotes significant differences as compared to pre-term born individuals at exhaustion. No differences between the groups were noted at rest*.

### Prooxidant/Antioxidant Balance

As detailed in Table 3. No baseline differences between the two groups were noted in neither of the measured oxidative stress nor antioxidant capacity markers (*p* > 0.05). Also, the hypoxia chemo-sensitivity test did not induce any significant between- or within-group changes in the measured markers (*p* > 0.05).

**Table 3:** Plasma values of the measured oxidative stress and antioxidant markers obtained prior (Pre) and immediately after (Post) the hypoxia chemo-sensitivity test in both groups.

	Pre-term		Full-term	
	Pre	Post	Pre	Post
AOPP (μmol ⋅ L^-1^)	153 ± 81	162 ± 86	143 ± 65	128 ± 36
MDA (μmol ⋅ L^-1^)	12.1 ± 3.5	13.8 ± 7.8	10.9 ± 4.1	9.7 ± 2.8
Nitrotyrosine (μmol ⋅ L^-1^)	39 ± 25	34 ± 22	40 ± 25	46 ± 37
SOD (mol ⋅ L^-1^ ⋅ min^-1^)	15.0 ± 4.1	15.2 ± 4.8	12.9 ± 3.8	13.1 ± 3.7
FRAP (μmol ⋅ L^-1^)	568 ± 105	585 ± 147	557 ± 85	563 ± 147
GPX (mol ⋅ L^-1^ ⋅ min^-1^)	96 ± 18	92 ± 17	96 ± 12	107 ± 31
Catalase (μmol⋅L^-1^ ⋅ min^-1^)	1.02 ± 0.72	0.91 ± 0.56	1.01 ± 0.47	0.85 ± 0.46

*Data are presented as mean ± SD. AOPP, advanced oxidation protein products; MDA, malondialdehyde; SOD, superoxide dismutase; FRAP, ferric-reducing antioxidant power; GPX, glutathione peroxidase*.

### Correlations

No significant correlations were observed between the measured MAP values in hypoxia and the calculated resting and exercise HVR values (*r*: -0.2 to 0.3; *p* > 0.5). Also, no correlations were noted between the hypoxia-related MAP reductions and the Δ
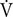
E, ΔSpO_2_ and ΔHR changes during the resting and exercise parts of the hypoxia chemo-sensitivity test nor between the baseline oxidative stress markers and HVR responses (*r*: -0.3 to 0.4; *p* > 0.4).

## Discussion

The primary aim of this study was to elucidate the effects of poikilocapnic hypoxia on HVR and exercise tolerance in prematurely born but otherwise healthy, physically active adults and compare their responses to those observed in their age as well as pulmonary and aerobic capacity matched counterparts born at full term. Thus, this is the first study to demonstrate that the previously observed reductions in HVR in untrained individuals can also be observed in active or “trained” prematurely born adult individuals which are more likely to be exposed to altitude during mountainous sporting activities. Additionally, we sought to investigate the potential differences in systemic oxidative stress between the two cohorts and further elucidate the relationship between oxidative stress levels and HVR modulation. Our findings indicate that the blunted resting HVR can still be observed in pre-terms following maturation. Interestingly, the reduced ventilatory response to acute normobaric hypoxia was not noted during moderate intensity exercise and moreover, the hypoxia-induced MAP reduction was comparable between the two cohorts. The obtained data also do not suggest that healthy, prematurely born adults would exhibit significantly higher systemic oxidative stress levels.

### Hypoxic Ventilatory Response

While research is limited, abnormal ventilatory responses to changes in ambient O_2_ availability have previously been reported in pre-term born infants (Katz-Salamon and Lagercrantz, 1994) and, recently, also in adults (Bates et al., 2014). These abnormalities might be a consequence of both perinatal periods of hypoxia and/or hyperoxia (Bavis, 2005). Perinatal hyperoxia, often employed in prematurity treatment, was suggested as the key underlying factor for the altered resting cardiorespiratory control (Bisgard et al., 2003) and carotid chemoreceptor dysfunction (Bates et al., 2014) observed in otherwise healthy pre-term born individuals. Our data lends further support to this notion since we show that in prematurely born individuals that underwent hyperoxic treatment at birth, the blunted resting HVR can still be observed after maturation (i.e., in adults). This abnormal ventilatory response might importantly compromise the hypoxic acclimatization ability of the pre-term born individuals. Especially, given that HVR plays a prominent role in the ventilatory acclimatization known to be a key determinant of, at least, short term acclimation/acclimatization to hypoxia (Powell et al., 1998; Teppema and Dahan, 2010). The fact that the prematurely born individuals, in addition to the blunted HVR, also demonstrated greater RH-related desaturation during the hypoxia sensitivity test could also negatively influence their acclimatization to prolonged hypoxic exposure and/or result in higher susceptibility to acute and chronic altitude sickness. Unfortunately, no study to date addressed this particular topic and further work on the consequences of chronic hypoxia in the pre-term born population is clearly needed. In contrast to the resting condition, our data indicate that the exercise HVR seems comparable between the pre-term and full-term born adults. Indeed, even a low-to moderate intensity exercise, as performed during the hypoxia chemo-sensitivity test, seems to abolish the blunted resting response in the active and healthy pre-term born individuals. This can be explained by the preponderant role of exercise-related respiratory drive (Forster et al., 2012; Duffin, 2014) that can override the blunted resting HVR and consequently enable optimal ventilatory response to hypoxic exercise. In this regard it is also prudent to note that aerobic capacity (e.g., training status) does not seem to importantly influence ventilatory responsiveness to hypoxia in humans (Sheel et al., 2006).

### Exercise Responses

As mentioned previously, reduced exercise capacity is commonly observed in pre-term born individuals (Rogers et al., 2005; Vrijlandt et al., 2006; Saigal et al., 2007; Lovering et al., 2013; Svedenkrans et al., 2013). This is in line with the present results where, despite a comparable 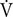
O_2peak_ and resting indices of pulmonary function the pre-term born individuals exhibited significantly lower MAP values during both, normoxic and hypoxic graded exercise tests (Figure 2.). Interestingly, while the consequences of prematurity on normoxic exercise capacity have been well studied, exercise tolerance during hypoxia received very little scientific attention. In contrast to their initial hypothesis both to-date studies (Duke et al., 2014; Farrell et al., 2015) that experimentally examined the effects of hypoxia on exercise performance did not find any significant hypoxia-related reduction in MAP. Even though the pulmonary gas exchange efficiency was not measured in the present study, it needs to be mentioned that both Duke et al. (2014) as well as Farrell et al. (2015) did not observe any differences in this potentially important exercise-limiting respiratory factor. The data of Farrell et al. (2015) also include an extremely interesting observation that whereas the MAP was significantly lower in the pre-term vs. the full-term individuals in normoxia, this difference was diminished during hypoxic exercise. While they initially hypothesized that hypoxia would exacerbate the MAP reductions, their somewhat paradoxical finding suggests that pre-term born individuals might actually tolerate hypoxia during exercise better when compared to their full-term born counterparts. One could thus speculate that the pre-term birth along with the associated medical and physiological consequences might precondition pre-term born individuals for subsequent hypoxic exposures as was recently demonstrated in a rodent model of neonatal hyperoxia (Goss et al., 2015).

Nevertheless, the fact that the observed hypoxia-related reductions in MAP were comparable between the two groups clearly suggests that acute hypoxia does not seem to compromise exercise capacity of the pre-term born individuals significantly more than it compromises the capacity of the full-term born adults. Also, our data indicate that regardless of the observed reduction in resting HVR in the pre-term group, the exercise ventilatory responses seem to be comparable between the two cohorts. The only ventilatory pattern difference observed between the groups was a higher tidal value at normoxic volitional exhaustion in the pre-term as compared to the full-term individuals. However, since both minute ventilation as well as respiratory frequency was similar between the groups and that no such difference was observed at volitional exhaustion in hypoxia we believe that this difference is physiologically negligible. It has to be emphasized again that these results are derived from a group of otherwise healthy and active pre-term and full-term born individuals that also display a relatively high aerobic capacity (i.e., mean 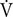
O_2peak_ = 49–53 mL ⋅ kg^-1^ ⋅ min^-1^). As mentioned in the introduction section, the reason for testing aerobically fit and active pre-term born individuals was that they are most likely to engage in activities at high-altitudes. Taken together, the data from the present and previous (Duke et al., 2014; Farrell et al., 2015) studies suggest that exercising in normobaric hypoxia seems to be well tolerated by healthy prematurely born adults. Nonetheless, the effects of prematurity *per se* on exercise hyperpnea, an extremely flexible and complex layered response comprising feed-forward, feed-backward and adaptive components (Mitchell and Babb, 2006; Forster et al., 2012), under both normoxic and hypoxic conditions warrants further scrutiny.

### Oxidative Stress and HVR Modulation

The third aspect of the present study was related to the redox-balance and its potential influence on HVR modulation. Although it is currently unclear whether the augmented systemic oxidative stress levels observed in the pre-term born infants (Robles et al., 2001; Buonocore et al., 2002) persist into adulthood recent evidence indicates that this might be the case (Filippone et al., 2012). Indeed, Filippone et al. (2012) found higher exhaled 8-isoprostane (oxidative stress marker) levels in pre-term as compared to the full-term born adolescents. While one could therefore expect that prematurely born adults also exhibit significantly elevated systemic oxidative stress levels (Martin et al., 2018) our analysis of a number of oxidative and antioxidant plasmatic markers of redox balance did not show any significant baseline differences between the two cohorts. Moreover, it is also plausible that ROS overproduction, potentially resulting from pre-term birth may not be detected within the plasma since the higher oxidative stress marker in pre-term adolescents was previously demonstrated in the exhaled breath and not in plasma (Filippone et al., 2012). These findings might also be importantly influenced by the selection criteria used in the present study (healthy, active, male survivors of prematurity with no chronic anatomical and clinical consequences). Based on the previously observed relation between oxidative stress and HVR in rodents (MacFarlane et al., 2008) and humans (Pialoux et al., 2009a,b) we also aimed to determine potential relation between the two in the pre-term born individuals. Nevertheless, no significant correlation was observed between the measured baseline oxidative/antioxidant markers and HVR responses. Also, the hypoxia sensitivity test did not provoke any measurable changes in the redox balance markers in either group even though oxidative stress changes were previously reported following such testing (Pialoux et al., 2009a). Additionally, both acute hypoxia (Magalhaes et al., 2005; Debevec et al., 2014) and exercise (Powers et al., 2011) have previously been show to independently augment oxidative stress (Debevec et al., 2017a). A possible explanation could be that such an acute and short-term (8 min) normobaric hypoxic exposure combined with a low-intensity exercise is not sufficient to importantly alter redox balance in otherwise healthy pre-term born individuals.

### Limitations and Methodological Considerations

While the present study provides novel insight into the hypoxic exercise tolerance of the otherwise healthy, active pre-term born adults, there are a few limitations we would like to acknowledge. Firstly, as with essentially all human studies on the topic, the independent effects of hyperoxia and prematurity are almost impossible to discern, since the vast majority of prematurely born infants are subjected to high inspired O_2_ levels. Secondly, the reported responses were obtained in a rather narrow age group (adults between 18 and 24 years of age) comprised of males only and prospective studies are warranted to investigate hypoxic exercise tolerance in both older and younger individuals of both sexes to provide additional insight into the potential maturation and sex-related effects. Thirdly, as we only tested acute effects of poikilocapnic hypoxia on ventilatory responses and exercise tolerance we cannot properly infer any valuable conclusions regarding the ability of pre-term born individuals to efficiently adapt to prolonged/chronic hypoxia. Accordingly, and especially given the blunted resting HVR observed in the present and other studies (Duke et al., 2014; Farrell et al., 2015), prospective investigations should also examine the ability and kinetics of the physiological acclimatization to prolonged hypoxic exposure in the pre-term born individuals. Also, it is of note that normobaric hypoxia has been employed in the present study as a surrogate for high altitude-related hypoxia. Since differential ventilatory (Savourey et al., 2003; Faiss et al., 2013) and oxidative stress (Ribon et al., 2016) responses between hypobaric and normobaric hypoxia have previously been demonstrated, this has to be taken into account. Accordingly, and especially given that all of the up-to-date studies, including this one, employed normobaric hypoxic exposures future well-designed studies investigating acute and chronic responses to terrestrial (e.g., hypobaric) hypoxia are warranted. Finally, our data show a rather high inter-individual variability for some oxidative stress markers, in particular nitrotyrosine. This is however usual for such assays (Ogino and Wang, 2007) as various exogenous modulators such as intake of nitrates and dietary antioxidants, physical fitness level and smoking could explain the oxidative stress plasma markers individual variations. By design, the participants were non-smokers and, moreover since the peak oxygen uptake, an established marker of physical fitness, was homogenous within our population, it is unlikely that these parameters play a major role in the observed variability. Potentially, the large variability in these markers could be related to the differences in exogenous nitrates and antioxidants intakes that were not controlled.

## Conclusion

In summary, the obtained data show that resting HVR, observed in pre-term born infants, remains blunted well into adulthood. However, the diminished resting HVR is overridden by exercise-related respiratory drive already at low-moderate exercise intensities. Importantly, the present results clearly indicate that hypoxia-related MAP reduction is comparable between the two groups and moreover, that healthy and active adult survivors of pre-term birth do not seem to exhibit significantly higher resting systemic oxidative stress levels as compared to their age and activity matched counterparts. Collectively, these findings suggest that healthy prematurely born adults tolerate exercise in hypoxic conditions well, and should, hence not be discouraged to engage in recreational or competitive activities in hypoxic conditions. Nevertheless, the observed blunted HVR and greater desaturation at rest in the pre-term vs. full-term born individuals warrant further investigation.

## Ethics Statement

The study protocol was pre-registered at ClinicalTrials.gov (NCT02780908), approved by the National Committee for Medical Ethics at the Ministry of Health of the Republic of Slovenia (0120-101/2016-2) and conducted according to the guidelines of the Declaration of Helsinki.

## Author Contributions

TD, VP, GM, MM, and DO conceived and designed the research. TD, MM, and DO conducted the experiments. TD, VP, GM, AM, and MM analyzed the data. TD wrote the manuscript. All authors read and critically revised the manuscript.

## Conflict of Interest Statement

The authors declare that the research was conducted in the absence of any commercial or financial relationships that could be construed as a potential conflict of interest.
